# The relationship between sleep problems and gaming addiction in adults

**DOI:** 10.1192/j.eurpsy.2022.2113

**Published:** 2022-09-01

**Authors:** C. Neily, M. Maalej, I. Gassara, R. Feki, N. Smaoui, L. Zouari, J. Ben Thabet, S. Omri, N. Charfi

**Affiliations:** Hedi Chaker hospital, Psychiatry Department, Sfax, Tunisia

**Keywords:** gaming, adults, Addiction, sleep

## Abstract

**Introduction:**

Prolonged exposure to Video games may have several negative cognitive and emotional consequences.However, a few investigations have explored the effects of video games addiction on sleep.

**Objectives:**

To study the effects of gaming addiction on sleep patterns in young adults

**Methods:**

We conducted a cross-sectional, descriptive and analytical study.Data were collected using a self-administered questionnaire on social networks targeting young adults between 18–40 years. We used the gaming addiction scale (GAS) in its validated Arabic short version. We also used the validated Arabic version of the Pittsburgh Sleep Quality Index (PSQI) to assess the sleep quality of our participants.

**Results:**

One hundred and nine participants were included. The mean age was 29.6 ± 10.3. Males accounted for 60.6% of the study population. The mean Gas score was 13.11± 6.08.According to the GAS,25.7% were addicted gamers. The mean PSQI score was 7.25± 3.15. A poor sleep quality pattern (score > 6) was found in 59.6% of the participants. We found that the GAS score was significantly correlated to the total score of PSQI( P=0.003). We also found that the group with poor sleep quality had higher GAS scores (p= 0.014). We found a correlation between the GAS score and the following components of the PSQI: subjective sleep quality ( p= 0.01), sleep disturbances (p=0.024) and the use of sleep-promoting medication ( p=0.046)

**Conclusions:**

Our study showed that video gaming behavior had a significant effect on sleep quality. This can have negative consequences on life quality, together with an impaired performance at awakening.

**Disclosure:**

No significant relationships.

